# Convolver Design and Convolve-Accumulate Unit Design for Low-Power Edge Computing †

**DOI:** 10.3390/s21155081

**Published:** 2021-07-27

**Authors:** Hsu-Yu Kao, Xin-Jia Chen, Shih-Hsu Huang

**Affiliations:** Department of Electronic Engineering, Chung Yuan Christian University, Taoyuan 32023, Taiwan; darren.kao.s@cycu.org.tw (H.-Y.K.); xinjiachen@cycu.org.tw (X.-J.C.)

**Keywords:** adder trees, convolution operations, dataflow, digital circuits, logic design, multiplications, partial product reduction

## Abstract

Convolution operations have a significant influence on the overall performance of a convolutional neural network, especially in edge-computing hardware design. In this paper, we propose a low-power signed convolver hardware architecture that is well suited for low-power edge computing. The basic idea of the proposed convolver design is to combine all multipliers’ final additions and their corresponding adder tree to form a partial product matrix (PPM) and then to use the reduction tree algorithm to reduce this PPM. As a result, compared with the state-of-the-art approach, our convolver design not only saves a lot of carry propagation adders but also saves one clock cycle per convolution operation. Moreover, the proposed convolver design can be adapted for different dataflows (including input stationary dataflow, weight stationary dataflow, and output stationary dataflow). According to dataflows, two types of convolve-accumulate units are proposed to perform the accumulation of convolution results. The results show that, compared with the state-of-the-art approach, the proposed convolver design can save 15.6% power consumption. Furthermore, compared with the state-of-the-art approach, on average, the proposed convolve-accumulate units can reduce 15.7% power consumption.

## 1. Introduction

Since AlexNet achieved outstanding achievements in the ImageNet Large-Scale Visual Recognition Challenge (ILSVRC), a lot of research teams have been devoted to the development of convolutional neural networks (CNNs) with well-known research advances such as ZFNet, GoogleNet, VGG, ResNet, etc. Owing to the increasing demand for real-time applications, an efficient dedicated hardware computation unit (i.e., a CNN accelerator) is required to support the calculations [1,2,3,4,5,6] in the inference process. Moreover, for edge devices, low power is also an important concern [7,8,9].

**Figure 1:**
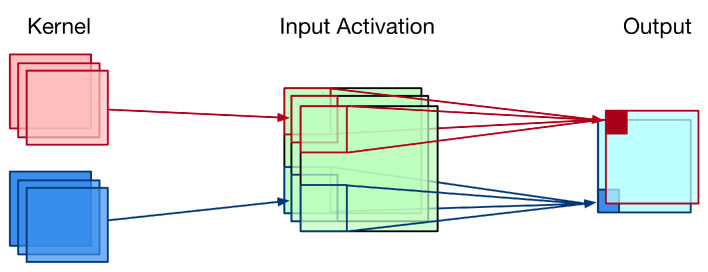
An illustration for the 2-D convolution. Adapted with permission from Ref [13] Copyright 2020 IEEE.

Convolution operation is a widely used technique in computer vision, signal processing, and image processing (such as edge detection and sharpening processing) [10,11,12]. Figure 1 gives an illustration for the two-dimensional (2-D) convolution. As shown in Figure 1, the 2-D convolution requires intensive data computations and high data throughputs. Thus, it is a challenge to realize the hardware circuit for 2-D convolution. It has been recognized that the 2-D convolution has a significant impact on the overall performance of a CNN accelerator.

Some methods [14,15,16,17,18,19] have been proposed to improve the data throughput of CNN accelerators. Bosi et al. [14] made use of the characteristic of FPGA for achieving 1 pixel/clock cycle. To solve heavy usage of BRAM, Bosi et al. [14] proposed single-window partial buffering (SWPB) to reduce the on-chip resource requirement. To improve SWPB, Zhang et al. [15] proposed multi-window partial buffering (MWPB) for balancing the resource usage between on-chip and off-chip. Thus, MWPB can be implemented on low-cost FPGA development boards. Sreenivasulu et al. [16] used a few multiplexers, ALU blocks, and control blocks to construct a pipeline 2-D convolution computing unit for saving hardware resource. Carlo et al. [17] used six pipeline stages to improve the utilization of FPGA. Moreover, Wong et al. [18] and Wang et al. [19] used the pipeline technique to reduce the latency of critical path in the 2-D convolution process.

Most previous works [14,15,16,17,18,19,20,21,22] focused on the dataflow optimization of 2-D convolution. Different from these previous works [14,15,16,17,18,19,20,21,22], in this paper, we study the optimization of underlying hardware circuit design for 2-D convolution. Note that a convolver is built by multipliers and adders. The kernel size determines the number of required multiplications and the number of required additions. The main idea behind our approach is to effectively integrate multiplications and additions and then to optimize the overall circuit architecture.

The proposed convolver design combines multipliers’ final additions and their corresponding adder tree to form a partial product matrix (PPM). Then, the reduction tree algorithm [23] is applied to reduce this PPM. As a result, the proposed approach can save a lot of carry propagation adders (CPAs) used for both final additions of multiplications and additions of the adder tree. The implementation results show that the proposed approach can save both circuit area and power consumption. Moreover, compared with the state-of-the-art approach [24], the proposed approach can also save one clock cycle (for performing final additions of multipliers and additions of adder tree) per convolution operation. In other words, the proposed approach can reduce the latency of convolution operation.

Note that Farrukh et al. [24] also treated the adder tree as a PPM for circuit optimization. However, in this state-of-the-art approach [24], the multipliers and the adder tree are still two separate computation components. On the other hand, some previous multiply-accumulate (MAC) designs [25,26,27,28] have tried to reduce the overheads caused by final additions of multiplications. However, since these MAC designs [25,26,27,28] assume that only one multiplier is used, their approaches cannot be directly applied to the design of 2-D convolver hardware circuit.

Chen and Huang [13] presented the first study for the optimization of underlying 2-D convolution hardware circuit design. Note that the proposed convolver design is a revised version of the previous work [13]. The main limitation of the previous work [13] is that it uses the Baugh–Wooley algorithm [29] for signed multiplications. Therefore, for long operands (i.e., large bit-width operands), the height of PPM is large. Different from the previous work [13], the proposed convolver design uses the modified Booth algorithm [30] for signed multiplications. Since the height of PPM is reduced by half, the proposed convolver design is more suitable for long operands. Note that longer operands can lead to a higher inference accuracy. Thus, the proposed convolver design is useful for applications requiring high accuracy.

Moreover, in the CNN accelerator, a convolve-accumulate unit is required to add up convolution results (from different channels). Note that the proposed convolver design can be adapted to become a convolve-accumulate unit. According to dataflows (including input stationary dataflow, weight stationary dataflow, and output stationary dataflow) described in [20], in this paper, we present two types of convolve-accumulate units to perform the accumulation of convolution results. The experiment results show that, on average, the proposed convolve-accumulate units can save 15.7% power consumption.

It is noteworthy to mention that, up until now, no special research attention has been paid to the optimization of underlying hardware circuit for the accumulation of convolution results (from different channels). Therefore, the proposed convolve-accumulate units are the first to deal with the optimization of underlying hardware circuit for the accumulation of convolution results.

The proposed approach can be integrated into existing CNN accelerators, e.g., [7,9,20,21,22]. However, to adopt the proposed approach, their processing element (PE) designs should be modified to deal with nine pixels at the same time. Note that, in the original PE designs of these CNN accelerators [7,9,20,21,22], their convolution operations are performed by multipliers and adders. If the proposed approach is adopted, their convolution operations are accelerated.

The contributions of our work are elaborated below:

1. We propose a low-power signed convolver hardware architecture for low-power edge computing. The proposed approach not only saves a lot of CPAs but also saves one clock cycle per convolution operation.
2. We propose two types of convolve-accumulate units to perform the accumulation of convolution results. The proposed approach is the first work to discuss the optimization of underlying hardware circuit for the accumulation of convolution results.

The rest of this paper is organized as follows. Section 2 presents the motivation to optimize the underlying hardware circuit design for 2-D convolution. In Section 3, we propose the architecture of signed 2-D convolver design. In Section 4, we present two types of convolve-accumulate units. The detailed experiment results are given in Section 5. Finally, we make some concluding remarks in Section 6.

## 2. Motivation

The basic operation of 2-D Convolution is to repeat a large number of multiplications and additions for calculations. Mathematically, 2-D convolution is to perform the summation of point-to-point multiplications as follows:

(1) $$Output=\sum_{k=0}^{n-1}Input_{k}*Weight_{k}$$

where Output is the output feature map, $Input_{k}$ is the input feature map, and $Weight_{k}$ is the weight of the filter. In terms of hardware implementation, for real-time applications, 2-D convolution needs to process multiple data and perform intensive computations at the same time. There is a need to increase the hardware parallelism to maintain or accelerate the overall hardware circuit performance.

If the 2-D convolution is performed by a MAC, the hardware cost is low. However, a lot of clock cycles are needed to obtain the convolution result. In contrast, the 2-D convolution can also be performed by multiple multipliers at the same time. Although the hardware cost is high, few clock cycles are needed to obtain the convolution result. For real-time applications, we need to choose this approach (i.e., multiple multipliers) to meet the high-performance requirement.

**Figure 2:**
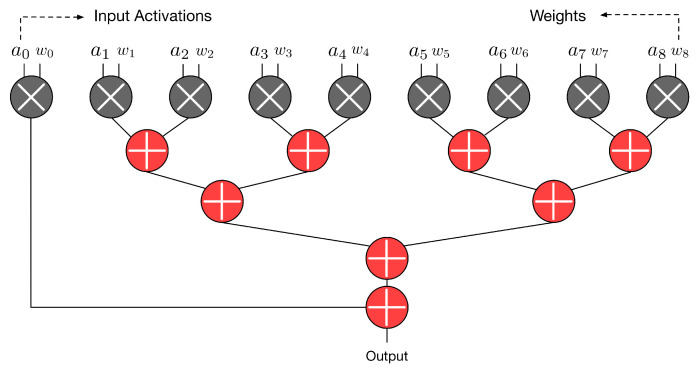
Operations of a 3 × 3 convolver.

Our objective is to optimize the underlying hardware circuit for 2-D convolution (i.e., to propose an optimized customized 2-D hardware convolver). The proposed hardware convolver performs N × N multiplications at the same time, where N × N is the kernel size. Figure 2 gives an example, in which the kernel size is assumed to be 3 × 3. In this example, nine multiplications and eight additions are required to complete a convolution. As shown in Figure 2, these nine multipliers are executed in parallel. Then, the outputs of nine multipliers form an adder tree to produce the convolution result.

**Figure 3:**
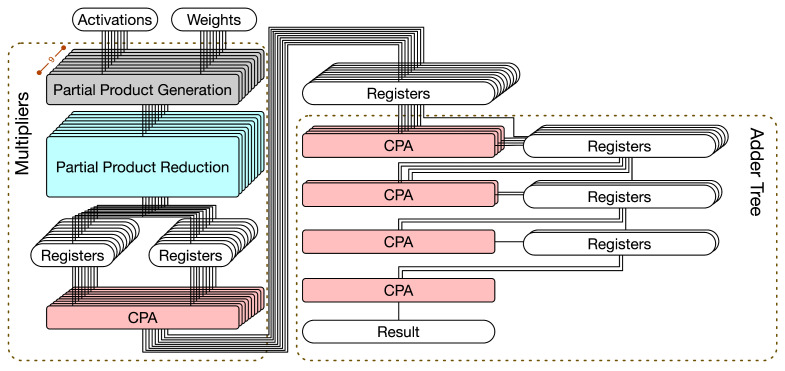
The straightforward hardware circuit implementation for the 3 × 3 convolver.

A multiplier usually consists of three steps (as shown in the left part of Figure 3): partial product generation (PPG), partial product reduction (PPR), and final addition. For signed multiplications, the PPG step often uses the modified Booth algorithm [30]. In the PPR step, the reduction tree algorithm, such as the Dadda tree approach [23] or the Wallace tree approach [31], is used to reduce the PPM. Finally, in the final addition step, a final adder is used to produce the final product. In practice, the final adder is often implemented by a CPA.

As shown in Figure 2, the adder tree is composed of adders. Note that the third step of a multiplier is also an adder (i.e., a final adder for the final addition of a multiplication). Thus, final adders of multipliers (i.e., the third step of multipliers) can also be thought of as a part of the whole adder tree. When considering the final adders of multipliers, the whole adder tree in Figure 3 has 17 adders (i.e., 8+9=17).

Figure 3 gives a straightforward hardware circuit implementation for the 3 × 3 convolver. This hardware circuit implementation includes two parts: multipliers and an adder tree. The details are elaborated on below.

1. Multipliers. In Figure 3, multipliers have three steps: PPG, PPR, and CPA (for final addition). Since the delay of PPG is small, both PPG and PPR are in the same pipeline stage. Owing to long carry chains, CPA requires one pipeline stage. As shown in Figure 3, 9 CPAs are used to perform final additions. The results are stored in nine registers.
2. Adder tree. In Figure 2, the height of adder tree is 4. Thus, as shown in Figure 3, the adder tree has four steps. In each step, CPAs are used to perform additions. Thus, the adder tree requires four pipeline stages. In the first stage, since the number of input registers is nine, which is an odd number, eight input registers are connected to four CPAs and one input register is directly connected to a register in the next stage (owing to the pipeline design); in the second stage, since the number of input registers is five, four input registers are connected to two CPAs and one input register is directly connected to a register in the next stage (owing to the pipeline stage); etc. As shown in Figure 3, eight CPAs are used in the adder tree.

In total, this straightforward hardware circuit implementation (displayed in Figure 3) uses 17 CPAs. It is also noteworthy to mention that registers are required between two successive pipeline stages. Since this hardware circuit implementation has six pipeline stages, six clock cycles are needed to complete a 3 × 3 convolution operation.

**Figure 4:**
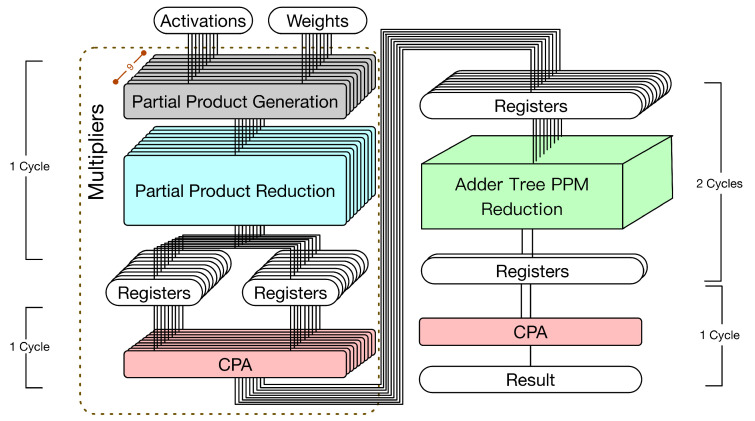
The state-of-the-art approach [24] (i.e., the hardware 3 × 3 convolver that treats the adder tree as a PPM).

In fact, as described in [24], the adder tree can also be represented by a PPM. Then, we can apply the reduction tree algorithm to reduce this PPM. As a result, a lot of CPAs can be saved. According to this observation [24], we can derive the corresponding hardware circuit implementation as shown in Figure 4. Note that this state-of-the-art hardware circuit implementation (i.e., Figure 4) still includes two parts: multipliers and an adder tree. The details are elaborated below.

1. Multipliers. The first pipeline stage performs both PPG and PPR. The second pipeline stage uses CPAs to perform final additions. As shown in Figure 4, nine CPAs are used to perform final additions.
2. Adder tree. The adder tree is represented by a PPM and then the reduction tree algorithm is applied to reduce this PPM. Note that the height of this PPM is nine. To reduce the height from nine to two, the Dadda tree approach [23] needs to perform the reduction process four times. (In the Dadda tree approach [23], the reduction process is controlled by a maximum-height sequence $d_{j}$, which is defined by $d_{1}=2$ and $d_{j+1}=floor\left(1.5\;d_{j}\right)$. In other words, the maximum-height sequence is $d_{1}=2,d_{2}=3,d_{3}=4,d_{4}=6,d_{5}=9$, etc. According to this maximum-height sequence, we know that the Dadda tree approach [23] needs to perform the reduction process four times to reduce the height from nine to two.) If this pipeline stage (i.e., all four reduction processes) is performed within a single clock cycle, a large clock period is needed (owing to a large combinational path delay). Thus, it is better to use two clock cycles to complete this pipeline stage. Finally, in the last pipeline stage, a CPA is used to produce the convolution result.

In total, this state-of-the-art hardware circuit implementation (displayed in Figure 4) uses 10 CPAs and has 4 pipeline stages. Since the third pipeline stage (i.e., the four reduction processes of the Dadda tree approach) uses two clock cycles, five clock cycles are needed to complete a 3 × 3 convolution operation. Compared with the straightforward hardware circuit implementation (displayed in Figure 3), which uses six clock cycles to complete a 3 × 3 convolution operation, this state-of-the-art hardware circuit implementation (displayed in Figure 4) can save one clock cycle (i.e., 6−5=1).

Although the state-of-the-art hardware circuit implementation (displayed in Figure 4) improves the straightforward approach (displayed in Figure 3), we still find that the 3 × 3 hardware convolver design can be further optimized. In the state-of-the-art hardware circuit implementation (displayed in Figure 4), the multipliers and the adder tree are still two separate computation components. In fact, final additions of multipliers can also be combined with additions of the adder trees. Then, we can use a single PPM to represent both final additions of multipliers and additions of the adder trees. By applying the reduction tree algorithm to this PPM, only one CPA is required. Based on this motivation, in the next section, we present the proposed hardware 3 × 3 convolver design.

## 3. Proposed Convolver Architecture

**Figure 5:**
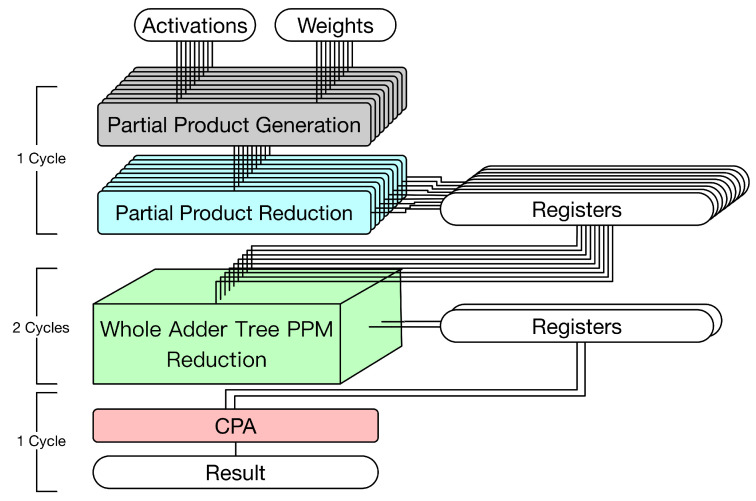
The proposed hardware circuit implementation for the 3 × 3 convolver.

The main idea behind the proposed approach is that final adders of multipliers can be thought of as a part of the whole adder tree. Thus, we can use a single PPM to represent both final additions of multipliers and additions of the adder trees. By applying the reduction tree algorithm [23] to this PPM, the overall circuit architecture can be further optimized. Figure 5 gives the proposed hardware 3 × 3 convolver design.

The proposed hardware 3 × 3 convolver design has three pipeline stages. In the first pipeline stage, both PPG and PPR of the multipliers are performed. In the second pipeline stage, we can use a single PPM to represent both final additions of multipliers and additions of adder tree. Then, the Dadda tree approach [23] is applied to reduce this PPM. Note that two clock cycles are required to complete this pipeline stage. Finally, in the third pipeline stage, a CPA is used to obtain the convolution result.

Note that the proposed hardware circuit implementation (displayed in Figure 5) only needs to use one CPA. In total, the proposed approach has three pipeline stages. Since the second pipeline stage uses two clock cycles, four clock cycles are needed to complete a 3 × 3 convolution operation. Compared with the state-of-the-art hardware circuit implementation (displayed in Figure 4), which uses five clock cycles to complete a 3 × 3 convolution operation, the proposed hardware circuit implementation (displayed in Figure 5) can save one clock cycle (i.e., 5−4=1). In the following, we elaborate on the details of the proposed hardware circuit implementation.

**Figure 6:**
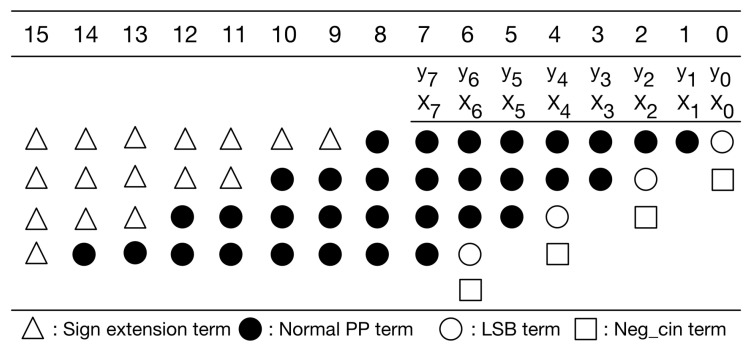
The typical PPM of radix-4 booth encoding.

**Figure 7:**
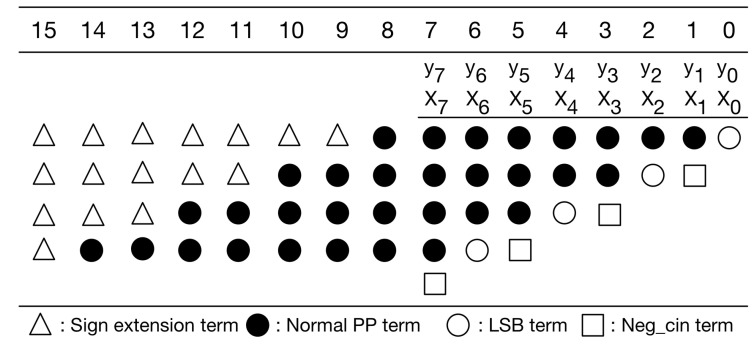
The modified PPM of radix-4 booth encoding.

Our PPM is a modification to the typical PPM of radix-4 booth encoding [30]. Without loss of generality, here we use 8-bit multiplication as an example for illustration. Figure 6 gives the typical PPM of radix-4 booth encoding for signed 8-bit multiplication. As displayed in Figure 6, the typical PPM (of radix-4 booth encoding) includes signed extension terms, normal partial product (PP) terms, least significant bit (LSB) term, and negative carry-in (Neg_cin) terms. It should be mentioned that the Boolean expressions of these terms, including signed extension terms, normal PP terms, LSB terms, and Neg_cin terms, have been elaborated in [32]. Moreover, according to [30], we can modify the Boolean expressions of both LSB terms and Neg_cin terms, and then, we can move each Neg_cin term to one higher bit position, as shown in Figure 7. Compared with previous PPMs [30,32], this PPM (i.e., Figure 7) does have any accuracy loss. Note that our following discussions and optimizations are based on this PPM (i.e., Figure 7).

**Figure 8:**
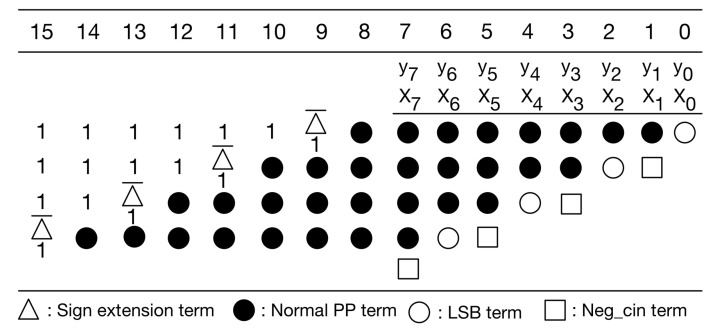
Replace sign extension terms.

**Figure 9:**
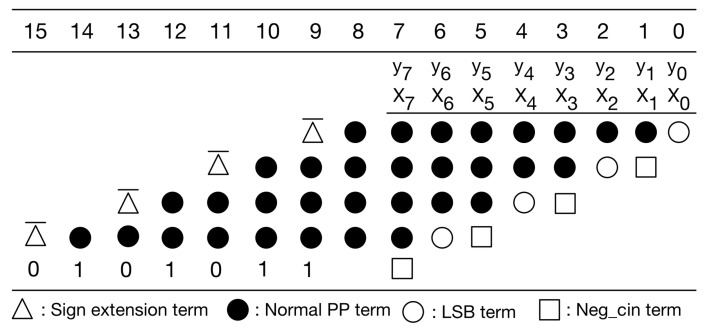
Our simplified PPM.

In Figure 7, the sign extension terms are either all 1 s or all 0 s. A large number of signed extension terms in each partial product can be replaced by an equal number of constant 1 s plus the inverse of signed extension term added to the least significant position, as displayed in Figure 8. Note that we can pre-compute the sum of these constants. The simplified PPM is given in Figure 9. As displayed in Figure 9, the string of constants is 0101011 (starting from the most significant bit).

**Figure 10:**
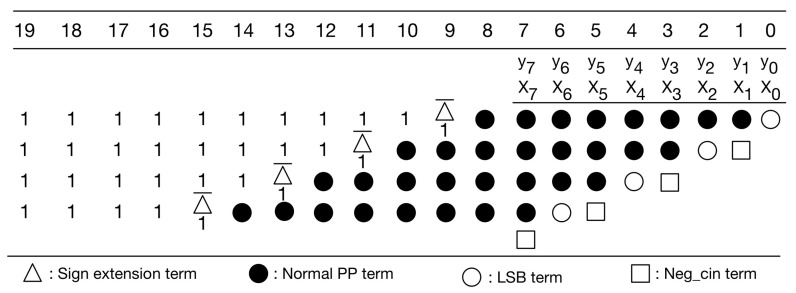
The modified PPM of radix-4 booth encoding with guard bits.

**Figure 11:**
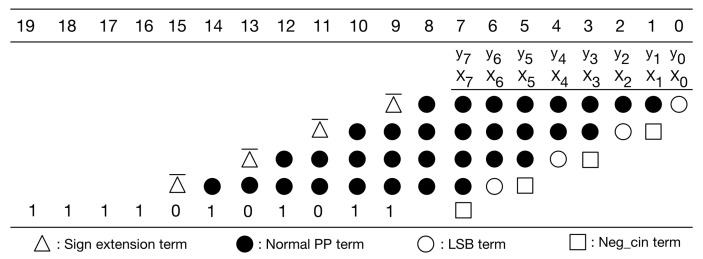
Our simplified PPM with guard bits.

**Figure 12:**
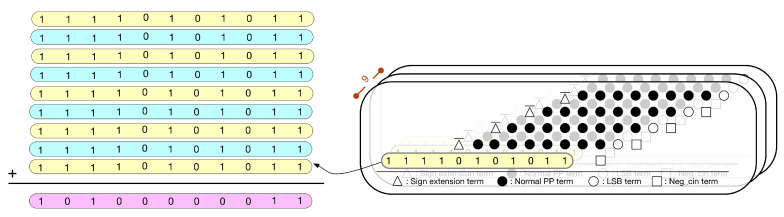
Pre-compute the sum of the constants of 9 PPMs.

Since the kernel size is 3 × 3, there are nine multiplications performed at the same time. In fact, nine multiplications correspond to nine PPMs. Thus, we can further pre-compute the sum of the constants in these nine PPMs. To avoid overflow, we need to use four guard bits. Figure 10 gives the PPM with guard bits. Figure 11 gives the simplified PPM with guard bits. Then, as shown in Figure 12, we can pre-compute the sum of the constants of these nine simplified PPMs. (Note that, since guard bits are used, here, the overflow can be omitted.) Figure 12 explains the pre-computation process. From Figure 12, we know that the string of pre-computed sum is 10100000011.

**Figure 13:**
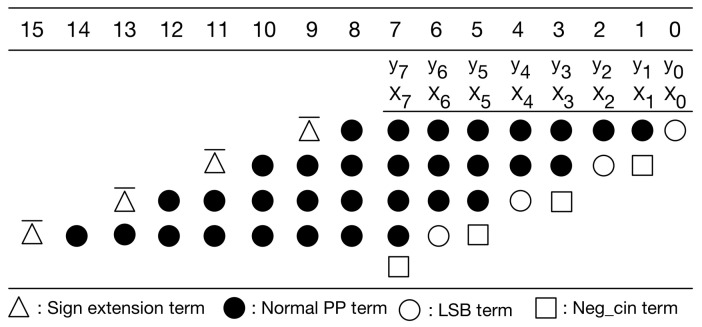
The PPM of each multiplication (in the first pipeline stage).

It is noteworthy to mention that the pre-computation process does not introduce any hardware resource usage. As a result, owing to the pre-computation process, both circuit area and power consumption can be reduced. Moreover, the pre-computed sum is directly used as a partial product of the PPM in the second pipeline stage. In other words, in the first pipeline stage, the PPR can ignore the constants. Therefore, in the first pipeline stage, for each multiplication, the PPR only needs to deal with the PPM shown in Figure 13. After the PPR is performed, each PPM (displayed in Figure 13) is reduced to become two partial products (i.e., two rows).

**Figure 14:**
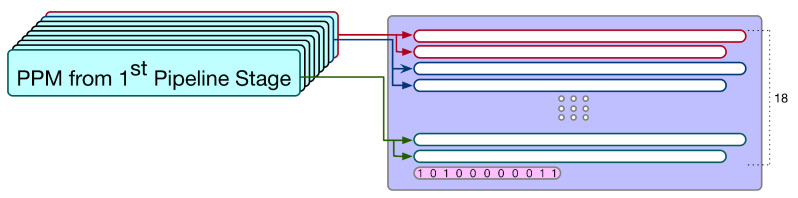
The PPM used in the second pipeline stage.

In the first pipeline stage, nine multiplications (i.e., nine PPMs) are performed at the same time. Thus, after the first pipeline stage is complete, we have 18 partial products (i.e., 9×2=18) for these 9 multiplications. As shown in Figure 14, by combining these 18 partial products with the pre-computed sum (i.e., pre-computed constant), we can derive a PPM with 19 partial products. Note that this PPM (as displayed in Figure 14) corresponds to the whole adder tree (including the final additions of multipliers).

Then, in the second pipeline stage, we use the Dadda tree approach [23] to reduce the height of this PPM (as displayed in Figure 14) from 19 to 2. (In fact, both the Wallace tree approach [31] and the Dadda tree approach [23] can be used for the reduction of our PPM. Compared with the Wallace tree approach [31], the Dadda tree approach [23] uses fewer counters for the reduction of our PPM. Thus, we adopt the Dadda tree approach [23].) Note that the Dadda tree approach needs to perform the reduction process six times. (The maximum-height sequence is $d_{1}=2,d_{2}=3,d_{3}=4,d_{4}=6,d_{5}=9,d_{6}=13,d_{7}=19$, etc. According to this maximum-height sequence, we know that the Dadda tree approach [23] needs to perform the reduction process six times to reduce the height from 19 to 2.) To reduce the clock cycle time, here, we use two clock cycles to complete the second pipeline stage (i.e., we use two clock cycles to reduce the height of this PPM from 19 to 2). After the Dadda tree approach is applied, the PPM is reduced to become two rows.

In the third pipeline stage, one CPA is used to perform the summation of the final two rows. As a consequence, the result of 3 × 3 convolution is produced.

In total, the proposed approach only needs to use four clock cycles to complete a 3 × 3 convolution operation. In other words, in the proposed hardware circuit implementation, the latency to perform one convolution operation is four clock cycles. On the other hand, in the proposed approach, convolution operations can also be performed in a pipeline way. If convolution operations are performed in a pipeline way, one convolution result can be produced per clock cycle.

Moreover, the proposed approach can greatly save both CPAs used for final additions of multiplications and CPAs used for the adder tree. In fact, the proposed approach only requires one CPA (for the final addition in the third pipeline stage to produce the result of 3 × 3 convolution). It is noteworthy to mention that the state-of-the-art approach (as shown in Figure 4) [24] requires 10 CPAs. Therefore, compared with the state-of-the-art approach [24], the proposed approach can save nine CPAs.

Our approach can be easily applied to the design of hardware 2 × 2 convolver. Since the kernel size is 2 × 2, there are four multiplications performed at the same time. Based on the same idea (proposed in this section), we can derive the PPMs used in each pipeline stage (for the hardware 2 × 2 convolver design). As a result, we can use three pipeline stages (i.e., four clock cycles) to complete a 2 × 2 convolution operation.

In fact, our approach can be generalized to the design of hardware N × N convolver. However, if N is greater than 3, the height of the PPM for the whole adder tree is greater than 19. As a result, the reduction of this PPM needs to use more than two clock cycles. In other words, if N is greater than 3, the second pipeline stage needs to use more than two clock cycles.

Finally, it is noteworthy to mention that, by filling zero values, we can replace a 2 × 2 kernel with a 3 × 3 kernel. Thus, instead of implementing a hardware 2 × 2 convolver, we also can use a hardware 3 × 3 convolver to perform 2 × 2 convolution operations.

## 4. Proposed Convolve-Accumulate Units

In a CNN accelerator, a convolve-accumulate unit is required to add up convolution results (from different channels). In this section, we modify the proposed hardware 3 × 3 convolver design to become a convolve-accumulate unit. According to dataflows (including input stationary dataflow, weight stationary dataflow, and output stationary dataflow) described in [20], we present two types of convolve-accumulate units for performing the accumulation of convolution results.

Here, we assume that the CNN accelerator is a 2-D systolic array. The objective of the 2-D systolic array is to obtain the accumulation of convolution results. The main function of each PE in the 2-D systolic array is to perform a 3 × 3 convolution operation. Therefore, each PE can be based on our hardware 3 × 3 convolver design. However, according to the specified dataflow, we need to make a corresponding modification to our hardware 3 × 3 convolver design. The detailed modifications are elaborated on below.

**Figure 15:**
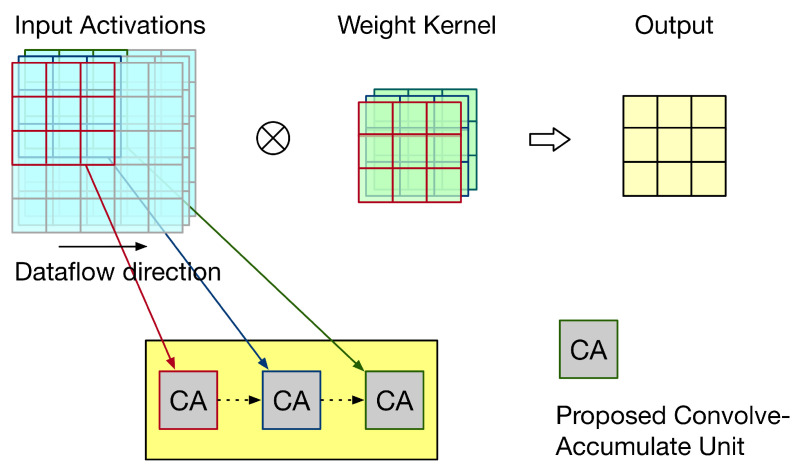
An illustration for weight stationary dataflow.

**Figure 16:**
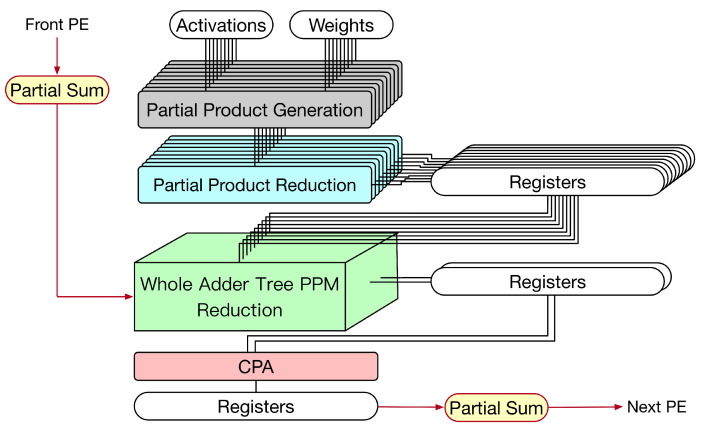
The proposed CA for weight stationary dataflow.

First, let us discuss the weight stationary dataflow. For weight stationary dataflow, weights are kept in the PE, and then, they are repeatedly used until all related computations have been completed. During processing, each PE sends the partial sum (i.e., the summation of its own convolution result and its front PEs’ convolution results) to its next PE for the accumulation of convolution results. Take the example shown in Figure 15 for illustration. In Figure 15, the convolution operation involves three channels and the systolic array has three PEs. Each PE stores one group of weights. Thus, each PE is responsible for the convolution of one channel. As the systolic array displayed in Figure 15, for each PE, its partial sum (i.e., the summation of its own convolution result and its front PEs’ convolution results) is sent to its next PE for the accumulation of convolution results. To reflect this dataflow, the PE needs to add its convolution result with its front PE’s partial sum. Thus, we say this PE is a convolve-accumulate unit (CA). Figure 16 gives the corresponding PE design, i.e., the corresponding CA design. As shown in Figure 16, for each PE, the partial sum of the front PE is treated as a partial product of its PPM in the second pipeline stage. (In fact, we can add the partial sum of the front PE and the pre-computed constant to become a single partial product (i.e., a single row). Then, the height of the whole adder tree PPM is still 19.) Therefore, for each PE, after the third pipeline stage is complete, its partial sum is produced.

Thus far, we assume that the dataflow is weight stationary dataflow. In fact, the proposed CA (displayed in Figure 16) can also be used for input stationary dataflow. For input stationary dataflow, input activations are kept in the PE, and then, they are repeatedly used until all related computations have been completed. During processing, each PE sends the partial sum to its next PE for the accumulation of convolution results. The proposed CA (displayed in Figure 16) can be used as a PE in the systolic array to reflect this dataflow.

**Figure 17:**
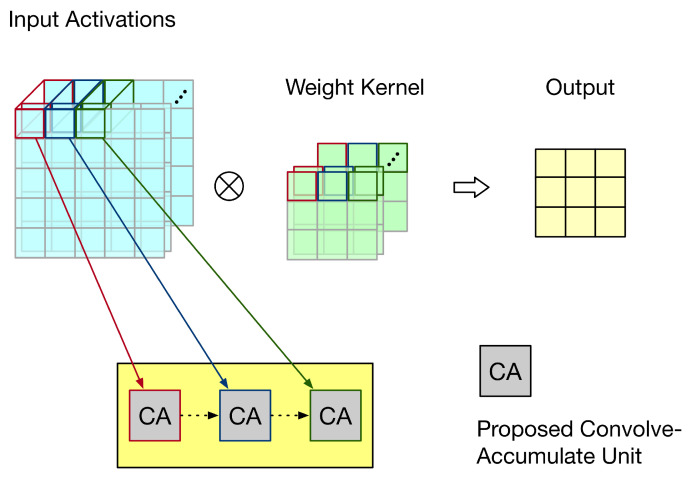
An illustration for output stationary dataflow.

**Figure 18:**
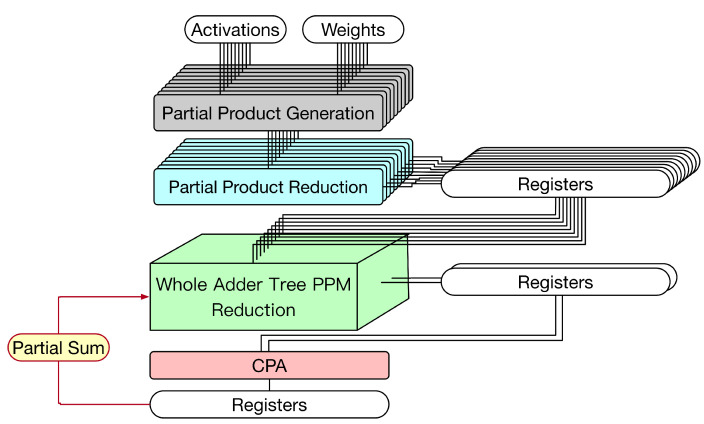
The proposed CA for output stationary dataflow.

Next, let us discuss the output stationary dataflow. For output stationary dataflow, partial sums are kept in the PE. Thus, the number of memory accesses for partial sums can be minimized. Take the example shown in Figure 17 for illustration. In Figure 17, the convolution operation involves three channels, the systolic array has three PEs, and the output has nine pixels. Thus, each PE is responsible for three output pixels. For each output pixel, since there are three channels, its PE needs to add up the convolution results of these three channels. Thus, after each convolution (for a channel) is performed, the convolution result must be added with the previous partial sum (i.e., the previous accumulated result) to produce the new partial sum. Then, this new partial sum is fed back to added with the next convolution result for further accumulation (until the convolutions of three channels have been performed). Note that, for each output pixel, the accumulation process has the same PE. Figure 18 gives the corresponding PE design, i.e., the corresponding CA design. As shown in Figure 18, for each PE, the partial sum of the front PE is treated as a partial product of its PPM in the second pipeline stage. Thus, for each PE, after the third pipeline stage is complete, its partial sum is produced.

## 5. Experimental Result

We used five CNNs, including Alexnet, vgg16, vgg19, resnet18, and resnet50, to evaluate the inference accuracy when the proposed convolver is used. Here, we assume that each input to the proposed convolver is in 16-bit fixed-point representation. Note that the evaluation is made in Pytorch framework. Table 1 gives the comparisons between the original inference accuracy (i.e., convolution operations are performed based on 32-bit floating point representation) and the inference accuracy of the proposed convolver (i.e., convolution operations are performed based on our 16-bit convolver). As shown in Table 1, we find that, no matter Top1 accuracy or Top 5 accuracy, our accuracy loss is at most 0.01%. In other words, in these five CNNs, our 16-bit convolver design can achieve almost the same inference accuracy as 32-bit floating point computation.

We also used TSMC 40 nm cell library to implement the proposed convolver design (i.e., Figure 5) and the proposed convolve-accumulate units (CA), including the proposed CA for weight stationary data flow (i.e., Figure 16) and the proposed CA for output stationary dataflow (i.e., Figure 18). For brevity’s sake, in the following, we use the notation CA-W to represent the proposed CA for weight stationary dataflow and the notation CA-O to represent the proposed CA for output stationary dataflow. In our experiments, we assume that the bit-width of each input is 16-bit. The kernel size is assumed to be 3 × 3 and 2 × 2, respectively. Then, we use Synopsys Design Compiler to synthesize these circuits with respect to different clock period constraints. Furthermore, we also used TSMC 40 nm cell library to implement the state-of-the-art convolver design (i.e., Figure 4) [24] and the state-of-the-art CA (i.e., the state-of-the-art convolver design [24] with an accumulator) for comparisons.

Table 2 and Table 3 tabulates the comparisons on circuit area and power consumption between the proposed convolver design and the state-of-the-art convolver design [24] with respect to different clock period constraints. In Table 2, the kernel size is assumed to be 3 × 3. As shown in Table 2, compared with the state-of-the-art convolver design [24], on average, the proposed convolver design can save both 12.8% circuit area and 16.1% power consumption (under the same clock period constraint). In Table 3, the kernel size is assumed to be 2 × 2. As shown in Table 3, compared with the state-of-the-art convolver design [24], on average, the proposed convolver design can save both 12.0% circuit area and 15.2% power consumption (under the same clock period constraint). Therefore, from Table 2 and Table 3, we know that the proposed convolver design can achieve both smaller circuit area and small power consumption (under the same clock period constraint) than the state-of-the-art convolver design [24].

Table 4 and Table 5 tabulate the comparisons on circuit area and power consumption between the proposed CA-W design and the state-of-the-art CA-W design [24] with respect to different clock period constraints. In Table 4, the kernel size is assumed to be 3 × 3. As shown in Table 4, compared with the state-of-the-art CA-W design [24], on average, the proposed CA-W design can reduce both 12.8% circuit area and 16.4% power consumption (under the same clock period constraint). In Table 5, the kernel size is assumed to be 2 × 2. As shown in Table 5, compared with the state-of-the-art CA-W design [24], on average, the proposed CA-W design can save both 12.2% circuit area and 15.1% power consumption (under the same clock period constraint). From Table 4 and Table 5, we know that the proposed CA-W design reduces both circuit area and power consumption.

Table 6 and Table 7 tabulate the comparisons on circuit area and power consumption between the proposed CA-O design and the state-of-the-art CA-O design [24] with respect to different clock period constraints. Table 6 assumes that the kernel size is 3 × 3. Compared with the state-of-the-art CA-O design [24], as displayed in Table 6, on average, the proposed CA-O design can save both 13.1% circuit area and 16.5% power consumption (under the same clock period constraint). Table 7 assumes that the kernel size is 2 × 2. Compared with the state-of-the-art CA-O design [24], as displayed in Table 7, on average, the proposed CA-O design can save both 11.9% circuit area and 14.8% power consumption (under the same clock period constraint). According to Table 6 and Table 7, we know that the proposed CA-O design reduces both circuit area and power consumption.

From these experimental results, we find that the proposed convolver design and the proposed convolve-accumulate units can save both circuit area and power consumption. According to Table 2 and Table 3, on average, the proposed convolver design can save both 12.4% circuit area and 15.6% power consumption (under the same clock period constraint). According to Table 4, Table 5, Table 6 and Table 7, on average, the proposed convolve-accumulate units can save both 12.5% circuit area and 15.7% power consumption (under the same clock period constraint).

**Figure 19:**
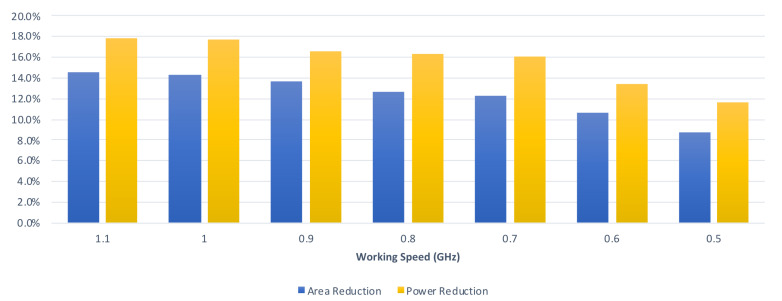
The reduction rates of the proposed CA design under different timing constraints.

**Figure 20:**
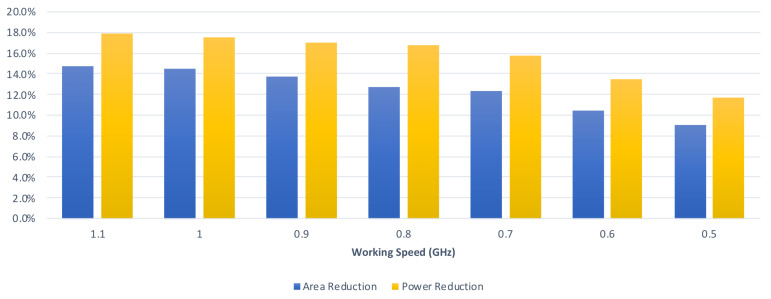
The reduction rates of the proposed CA-W design under different timing constraints.

**Figure 21:**
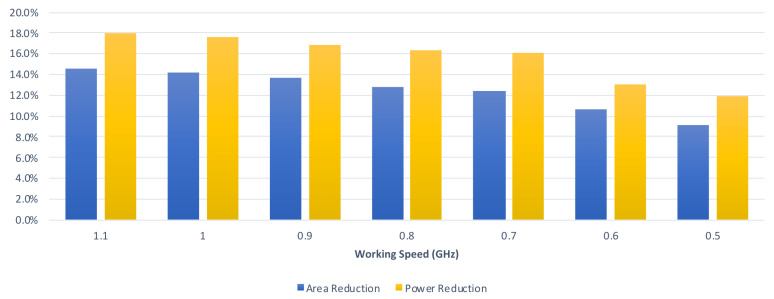
The reduction rates of the proposed CA-O design under different timing constraints.

Finally, we perform an analysis of the relation between the reduction rate and the clock period constraint. From Table 2 and Table 3, we can derive Figure 19, which displays the average reduction rates of the proposed convolver design in the graphical form with respect to different clock period constraints. From Table 4 and Table 5, we can derive Figure 20, which displays the average reduction rates of the proposed CA-W design in graphical form with respect to different clock period constraints. From Table 6 and Table 7, we can derive Figure 21, which displays the average reduction rates of the proposed CA-O design in graphical form with respect to different clock period constraints. In Figure 19, Figure 20 and Figure 21, the blue chart and the orange chart denote the reduction rate on circuit area and the reduction rate on power consumption, respectively. We find that the reduction rate is inversely proportional to the working speed. The reason is that the critical path of the proposed approach is shorter than that of the state-of-the-art approach [24]. Thus, it is easier for the proposed approach to satisfy the clock period constraint. As a result, the proposed approach can achieve a higher reduction rate in a higher working speed. Note that high performance is the trend of CNN accelerators. Therefore, the proposed approach is promising.

## 6. Conclusions

This paper presents a low-power signed convolver hardware architecture for low-power edge computing. The main feature of our approach is that we combine all multipliers’ final additions and the corresponding adder tree to form a PPM. By applying the Dadda tree approach to reduce this PPM, a lot of CPAs can be saved. Experiment results show that, compared with the state-of-the-art convolver design, on average, the proposed approach can save both 12.4% circuit area and 15.6% power consumption.

The proposed convolver design can be easily adapted for different dataflows. This paper presents two types of CAs for performing the accumulation of convolutions, including CA-W for both weight stationary dataflow and input stationary dataflow and CA-O for output stationary dataflow. Compared with the state-of-the-art CA design, the experimental data also show that, on average, the proposed approach can save both 12.5% circuit area and 15.7% power consumption. The proposed CAs are the first to deal with the optimization of underlying hardware circuit for the accumulation of convolution results.

We also find that the proposed approach can achieve a larger improvement within a tighter clock period constraint. Since high performance is the trend in CNN accelerators, we believe the proposed approach is promising.

## Figures and Tables

**Table 1:** Comparisons on the inference accuracy.

CNN	Convolution Approach	Top1 Accuracy	Top5 Accuracy
Alexnet	32-bit Floating Point	55.46%	78.44%
	Ours	55.46%	78.44%
vgg16	32-bit Floating Point	70.15%	89.48%
	Ours	70.15%	89.47%
vgg19	32-bit Floating Point	70.95%	89.98%
	Ours	70.95%	89.97%
resnet18	32-bit Floating Point	68.47%	88.30%
	Ours	68.46%	88.30%
resnet50	32-bit Floating Point	74.60%	92.22%
	Ours	74.60%	92.21%

**Table 2:** Comparisons on different 3 × 3 convolver designs.

Working Speed (GHz)	Area (μm${}^{2}$)			Power (mW)		
	State-of-the-Art [24]	Ours	Reduction Rate	State-of-the-Art [24]	Ours	Reduction Rate
1.1	14,890.91	12,683.56	14.8%	13.5477	11.0914	18.1%
1.0	14,824.10	12,640.01	14.7%	12.2879	10.0703	18.0%
0.9	14,736.78	12,629.81	14.3%	10.9986	9.0954	17.3%
0.8	14,516.56	12,611.21	13.1%	9.6016	7.9655	17.0%
0.7	14,418.35	12,594.65	12.6%	8.3408	6.9642	16.5%
0.6	14,140.97	12,583.77	11.0%	6.9206	5.9813	13.6%
0.5	13,763.81	12,524.80	9.0%	5.6360	4.9508	12.2%

**Table 3:** Comparisons on different 2 × 2 convolver designs.

Working Speed (GHz)	Area (μm${}^{2}$)			Power (mW)		
	State-of-the-Art [24]	Ours	Reduction Rate	State-of-the-Art [24]	Ours	Reduction Rate
1.1	6839.29	5866.86	14.2%	6.3007	5.1959	17.5%
1	6780.41	5834.88	13.9%	5.7157	4.7295	17.3%
0.9	6707.83	5844.18	12.9%	5.0762	4.2681	15.9%
0.8	6632.08	5822.18	12.2%	4.4684	3.7677	15.7%
0.7	6586.72	5797.91	12.0%	3.8827	3.2802	15.5%
0.6	6457.9	5791.11	10.3%	3.2372	2.8116	13.1%
0.5	6254.01	5727.83	8.4%	2.5986	2.3079	11.2%

**Table 4:** Comparisons on different 3 × 3 CA-W designs.

Working Speed (GHz)	Area (μm${}^{2}$)			Power (mW)		
	State-of-the-Art [24]	Ours	Reduction Rate	State-of-the-Art [24]	Ours	Reduction Rate
1.1	15,246.13	12953	15.0%	13.7132	11.1473	18.7%
1	15,091.43	12892.67	14.6%	12.3171	10.1055	18.0%
0.9	14,970.93	12875.62	14.0%	11.0293	9.1002	17.5%
0.8	14,783.05	12859.31	13.0%	9.7087	8.0209	17.4%
0.7	14,720.9	12827.29	12.9%	8.4305	7.0136	16.8%
0.6	14,375.49	12796.85	11.0%	7.0029	6.0083	14.2%
0.5	14,030.52	12707.89	9.4%	5.6831	4.9902	12.2%

**Table 5:** Comparisons on different 2 × 2 CA-W designs.

Working Speed (GHz)	Area (μm${}^{2}$)			Power (mW)		
	State-of-the-Art [24]	Ours	Reduction Rate	State-of-the-Art [24]	Ours	Reduction Rate
1.1	6996.53	5980.94	14.5%	6.4318	5.3231	17.2%
1	6952.55	5960.75	14.3%	5.8137	4.8219	17.1%
0.9	6884.74	5955.31	13.5%	5.2095	4.3463	16.6%
0.8	6816.02	5962.11	12.5%	4.5676	3.8334	16.1%
0.7	6727.34	5924.69	11.9%	3.9671	3.3793	14.8%
0.6	6559.28	5914.26	9.8%	3.2894	2.873	12.7%
0.5	6403.47	5851.21	8.6%	2.6791	2.381	11.1%

**Table 6:** Comparisons on different 3 × 3 CA-O designs.

Working Speed (GHz)	Area (μm${}^{2}$)			Power (mW)		
	State-of-the-Art [24]	Ours	Reduction Rate	State-of-the-Art [24]	Ours	Reduction Rate
1.1	15,267.94	12976.36	15.0%	13.7348	11.1621	18.7%
1	15,178.36	12935.76	14.8%	12.3763	10.1368	18.1%
0.9	15,098.75	12954.36	14.2%	11.0646	9.1284	17.5%
0.8	14,947.02	12934.17	13.5%	9.7612	8.0641	17.4%
0.7	14,863.56	12894.26	13.2%	8.4959	7.0349	17.2%
0.6	14,547.17	12879.06	11.5%	7.0223	6.0232	14.2%
0.5	14,200.71	12826.9	9.7%	5.7555	5.0217	12.7%

**Table 7:** Comparisons on different 2 × 2 CA-O designs.

Working Speed (GHz)	Area (μm${}^{2}$)			Power (mW)		
	State-of-the-Art [24]	Ours	Reduction Rate	State-of-the-Art [24]	Ours	Reduction Rate
1.1	7193.81	6175.31	14.2%	6.4788	5.3626	17.2%
1	7087.04	6121.1	13.6%	5.8927	4.8894	17.0%
0.9	7063.91	6132.67	13.2%	5.2329	4.3888	16.1%
0.8	6954.82	6122.91	12.0%	4.6139	3.9034	15.4%
0.7	6908.55	6100.91	11.7%	3.9796	3.3863	14.9%
0.6	6751.83	6082.32	9.9%	3.2936	2.8995	12.0%
0.5	6576.97	6017.23	8.5%	2.6823	2.3839	11.1%

## Data Availability

The data used to support the findings of this study are included in this paper.
